# Retraction: Pre-operative Aortic Anatomic Features as Predictors of Clinical Outcomes Following Endovascular Repair of Abdominal Aortic Aneurysm: A Retrospective Cohort Study

**DOI:** 10.7759/cureus.r79

**Published:** 2023-10-26

**Authors:** Oluwanifemi O Akintoye

**Affiliations:** 1 Department of Public Health and Primary Care, University of Cambridge, Cambridge, GBR

This article has been retracted and its contents removed at the request of the author due to the use of unapproved data. The data was obtained through a data agreement process between the Cambridge Clinical School Research Operation Office and Imperial College London. Data use was approved only for a Master's dissertation project, not publication. Further, the data provider and the dissertation supervisor did not review or endorse the publication of this work prior to submission. As a result, the author has requested that the article be retracted and its contents removed and the journal has agreed.

